# The microbiota: a crucial mediator in gut homeostasis and colonization resistance

**DOI:** 10.3389/fmicb.2024.1417864

**Published:** 2024-08-06

**Authors:** Yiding Chen, Ling Xiao, Min Zhou, Hu Zhang

**Affiliations:** ^1^Department of Gastroenterology, West China Tianfu Hospital, Sichuan University, Chengdu, China; ^2^Department of Gastroenterology, West China Hospital, Sichuan University, Chengdu, China; ^3^Center for Inflammatory Bowel Disease, West China Hospital, Sichuan University, Chengdu, China

**Keywords:** microbiota, gut homeostasis, enteric infections, mucosal immunity, colonization resistance

## Abstract

The gut microbiota is a complex and diverse community of microorganisms that colonizes the human gastrointestinal tract and influences various aspects of human health. These microbes are closely related to enteric infections. As a foreign entity for the host, commensal microbiota is restricted and regulated by the barrier and immune system in the gut and contributes to gut homeostasis. Commensals also effectively resist the colonization of pathogens and the overgrowth of indigenous pathobionts by utilizing a variety of mechanisms, while pathogens have developed strategies to subvert colonization resistance. Dysbiosis of the microbial community can lead to enteric infections. The microbiota acts as a pivotal mediator in establishing a harmonious mutualistic symbiosis with the host and shielding the host against pathogens. This review aims to provide a comprehensive overview of the mechanisms underlying host-microbiome and microbiome-pathogen interactions, highlighting the multi-faceted roles of the gut microbiota in preventing enteric infections. We also discuss the applications of manipulating the microbiota to treat infectious diseases in the gut.

## 1 Introduction

An enormous number of microbes, collectively termed gut microbiota, colonize the human gastrointestinal (GI) tract, including bacteria, viruses, fungi, archaea, and protozoa. Bacteria achieve the highest density, with an estimated more than 10^14^ bacteria in the gut (de Vos et al., 2022). The negative impact of microorganisms has long been the primary focus, but there is a growing interest in exploring their potential benefits for human health. These beneficial roles include supporting the maturation of the immune system, producing beneficial metabolites, promoting food digestion and vitamin production, enhancing gut barrier function, and resisting the colonization of pathogens (Dey et al., 2021).

Bacterial gastroenteritis poses a significant threat to global public health, and the microbiota is tightly linked to the infection in the gut (GBD, 2019 Antimicrobial Resistance Collaborators, 2022). Infectious enteritis occurs when pathogens invade the intestine and elicit a response from the immune system (Sarkar et al., 2024). The microbiota is recognized by the host as a foreign entity, making it a potential source of infection. In a healthy state, a harmonious interaction is maintained between the gut and the microbiota. The commensal microbiota, which dominates and occupies nearly all areas of the gut, is restricted and regulated by the host and serves as an inevitable symbiont for the host (Kim et al., 2017). There exists another interaction between microbiota and pathogens in the gut. The microbiota efficiently impedes pathogen colonization by deploying multiple mechanisms to prevent infection. However, during dysbiosis, some beneficial commensals can exert adverse effects, and pathogenic microbes have counterstrategies to break the defense from the microbiota and host (Ducarmon et al., 2019; Caballero-Flores et al., 2023). Therefore, gut microbiota acts as a significant mediator in maintaining gut homeostasis and protecting against pathogen invasion. The current advancement of research and technologies supply mechanistic insights into host-microbiome and microbiome-pathogen interactions, enhancing our understanding of these dynamic interactions and promoting the development of innovative strategies to reduce the incidence and severity of enteric infections.

**Figure 1:**
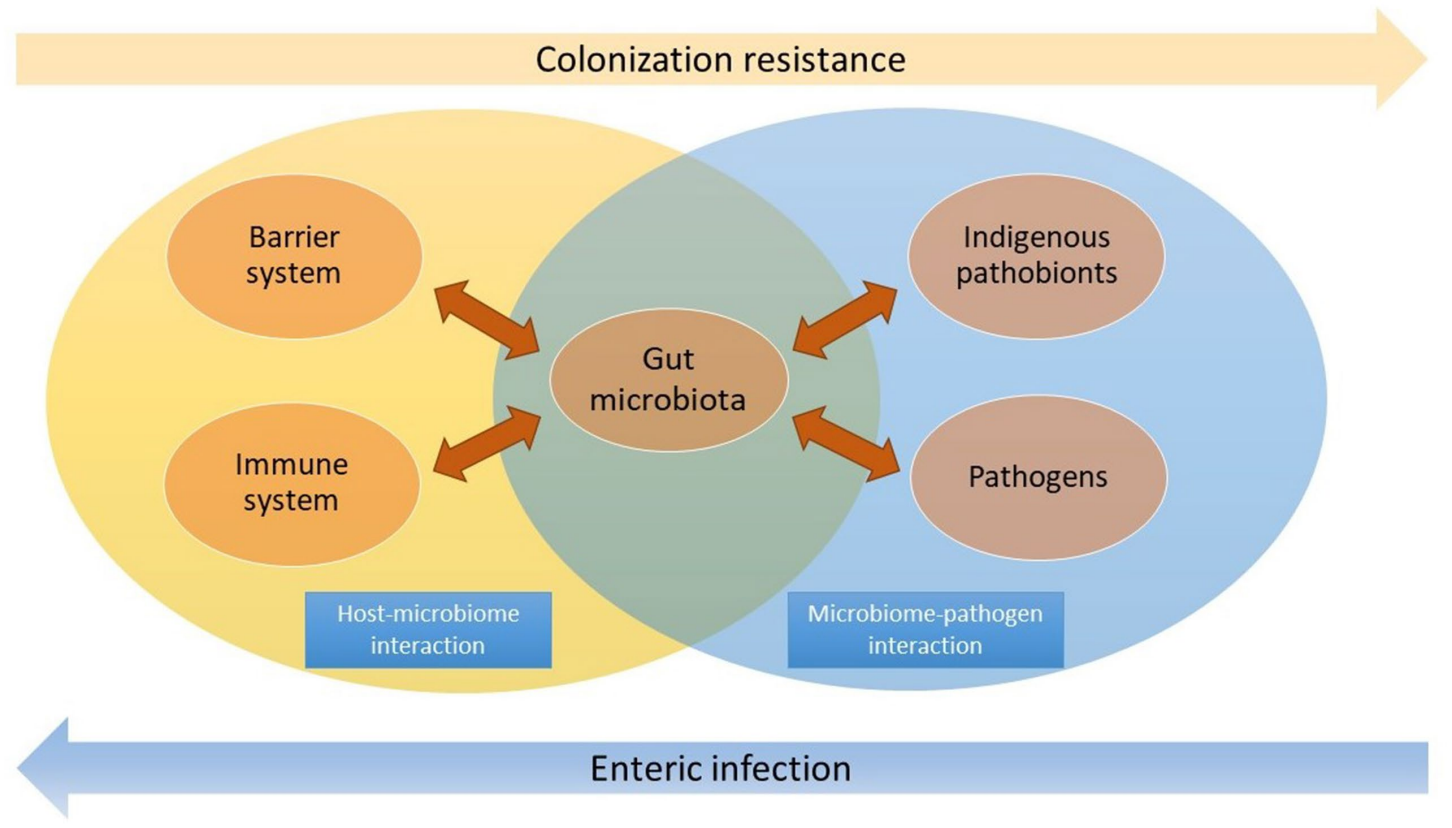
The microbiota acts as a vital mediator in the host-microbiome and microbiome-pathogen interactions. Commensal microbiota interacts with the barrier and immune system in the gut, while it also effectively resists pathogen colonization and overgrowth of indigenous pathobionts. The dysbiosis may lead to enteric infection.

This review systematically elucidates the multi-faceted roles of microbiota as both a foreign entity and a symbiont in the gut. The commensal microbiota must be modulated by the host and maintain a balanced gut environment. It also aids the host in effectively resisting the invasion and colonization of pathogens (Figure 1). By delving into the intricate roles of gut microbiota, we aim to facilitate the comprehension and promotion of the mutually beneficial relationship between the host and microbiota to prevent and manage infectious diseases in the gut.

## 2 Composition of microbiota

The normal gut microbial community, known as the commensal microbiota, typically consists of six significant phyla, including *Firmicutes*, *Bacteroidetes*, *Actinobacteria*, *Proteobacteria*, *Fusobacteria*, and *Verrucomicrobia*, among which the first two phyla make up over 90% of the bacterial population in the colon while the others are present in lower abundance (Hou et al., 2022). The total density of bacteria is higher in the colon than in the small intestine. Members of *Firmicutes*, *Proteobacteria*, and *Actinobacteria* are the prominent residents of the small intestine (Ruigrok et al., 2023).

The *Bacteroidetes* phylum includes both anaerobic and aerobic, non-spore-forming, Gram-negative, rod-shaped bacteria that colonize the intestinal tract. The *Bacteroides* genus is one of the most predominant groups in the intestine. It has essential metabolic functions and maintains a beneficial relationship in the intestinal lumen, but some members can become pathogenic if they disseminate (Rajilić-Stojanović and de Vos, 2014). For example, *Bacteroides fragilis* is typically found in the lower GI tract and has beneficial effects. However, it is also commonly isolated from abdominal abscesses and bloodstream infections if the intestinal mucosa is perforated and traversed (Mazmanian and Kasper, 2006; Wexler and Wexler, 2007).

The *Firmicutes* phylum is composed of anaerobic bacteria that are predominantly Gram-positive and can produce endospores. These endospores are durable, dormant structures that allow the bacteria to remain viable in challenging environments and reactivate when conditions become more favorable. The class *Clostridia* encompasses a wide range of bacteria with different clusters. While some clusters, such as *XIVa* and *IV*, exert beneficial effects by producing butyrate and supporting intestinal health and immune balance, others, like cluster *I*, *Clostridium perfringens*, *Clostridium tetani*, and *Clostridium difficile,* are potential pathogens responsible for enteric infection (Kim et al., 2017). The *Firmicutes* phylum also involves the *Bacilli* class, which includes clinically significant oxygen-tolerant pathobionts such as *Enterococcus* species and *Streptococcus* species (Taur et al., 2012, 2013). These bacteria are generally present in low abundance but can become harmful during intestinal dysbiosis.

The *Actinobacteria* phylum comprises aerobic and anaerobic bacteria (Rajilić-Stojanović and de Vos, 2014). *Bifidobacteria* species are among the predominant genera in this phylum. They are recognized for their probiotic properties, including mechanisms such as competitive exclusion, bile salt hydrolase activity, immune modulation, and adherence to the mucus or intestinal epithelium (Kim et al., 2017), thus protecting the gut against potential infections caused by harmful bacteria.

The *Proteobacteria* phylum encompasses a wide array of Gram-negative bacteria, many of which are facultative anaerobes. An elevated presence of *Proteobacteria* in the gut microbiota may signify an imbalance and a heightened risk of illness (Shin et al., 2015). The family *Enterobacteriaceae* within the *Gammaproteobacteria* class includes pathogens such as *Escherichia coli* and *Klebsiella* species (Taur et al., 2012, 2013). These pathogens are typically in low abundance but can become dominant in the intestinal environment during dysbiosis.

The *Fusobacteria* phylum is a type of Gram-negative anaerobic bacilli that is recognized as an opportunistic pathogen due to its frequent isolation from anaerobic samples in various infections; its role as a cancer-causing member of the microbiota is still being uncovered (Brennan and Garrett, 2019; Harrandah, 2023). The *Verrucomicrobiota*, a phylum of Gram-negative bacteria, are mucin-degrading bacteria found in the intestinal mucosa. This phylum of bacteria can help maintain intestinal health and glucose homeostasis (Oren and Garrity, 2021; Jian et al., 2023).

Additionally, fungi are crucial gut microbiota components, influencing immune responses and local inflammation. The most extensively researched fungi in the gut microbiota include *Candida*, *Saccharomyces*, *Malassezia*, and *Cladosporium*. The gut microbiota also comprises viruses, phages, and archaea, with *Methanobrevibacter smithii* being the predominant archaea species (Hou et al., 2022).

In general, the gut microbiota is a complex and dynamic community of microorganisms colonizing the GI tract. The diversity and balance of the gut microbiota are critical for maintaining intestinal health and preventing diseases. The altered composition or distribution of gut microbiota could create opportunities for pathobionts to thrive and colonize in the gut, thus ultimately leading to infections (Sheppard, 2022). Notably, there is no strict boundary between beneficial and harmful microbes. The beneficial microbiota present in most situations has the potential to transform into harmful pathogens during dysbiosis quickly.

## 3 Crosstalk between gut and microbiota

**Figure 2:**
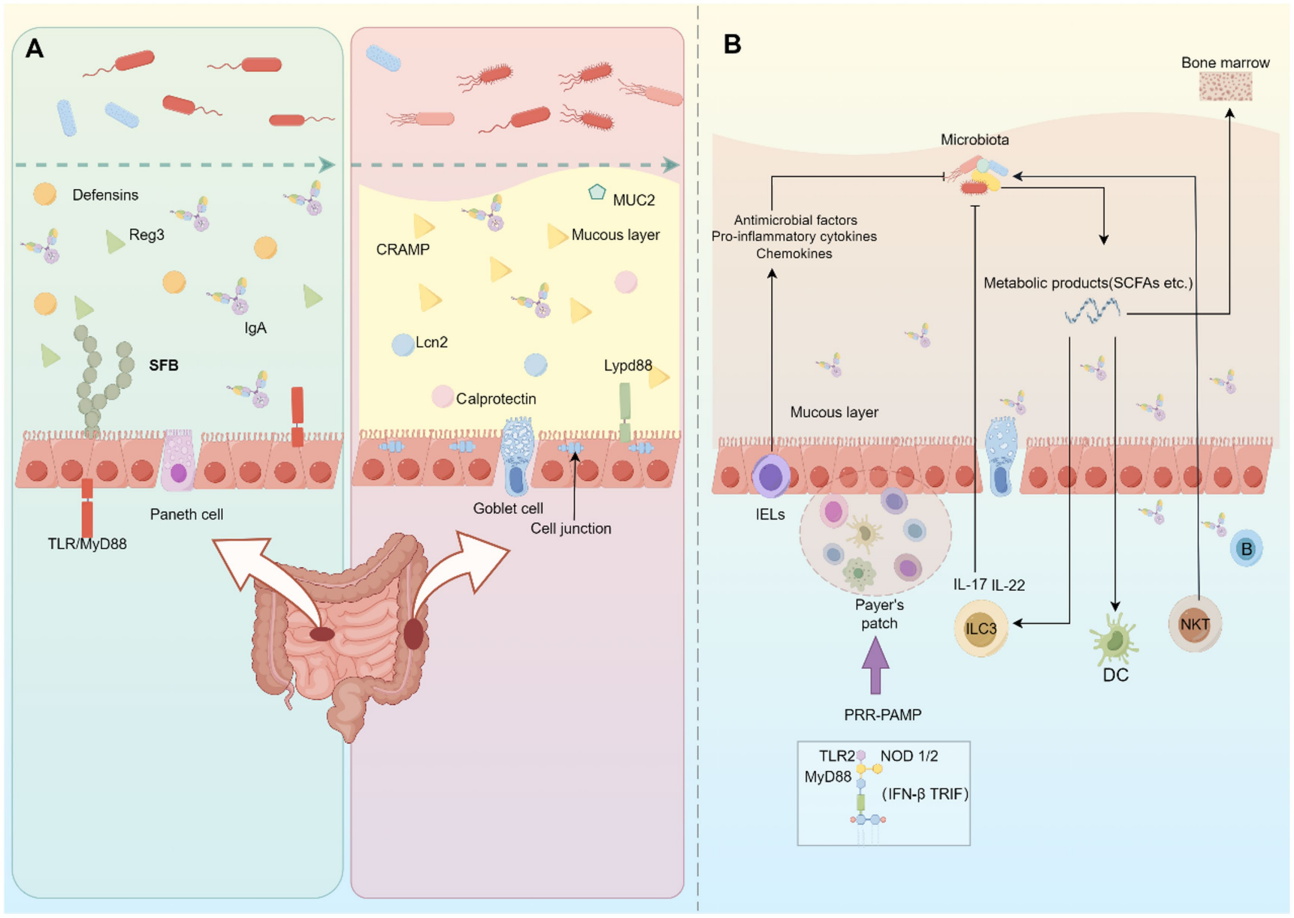
Host-microbiome interaction in the gut. **(A)** In the small intestine, chemical barriers mainly contribute to separating the gut microbiota and intestinal epithelial cells. Immune cells in the lamina propria regulate the generation of these chemical barriers. In the large intestine, the mucous layer and cell junctions hamper pathogen invasion in the gut. **(B)** The host immunity can recognize and respond to the presence of gut microbiota. This interaction involves various immune cells and cytokines, and the commensal microorganisms and their metabolites can impact the development and function of mucosal immunity.

While the gut microbiota has been shown to benefit the host, it is important to note that the host recognizes the microbiota as a foreign entity. To prevent excessive defensive responses, the barrier system and mucosal immune system in the gut contribute to maintaining the balance between the host and commensal microbes (Figure 2).

### 3.1 Barrier-microbiome interaction

Intestinal epithelial cells (IECs) are crucial in establishing physical and chemical barriers to maintain the separation of microbiota and immune cells (Maynard et al., 2012). Specifically, the barrier mechanisms vary between the small intestine and the colon.

#### 3.1.1 Chemical barriers

The small intestine possesses fewer goblet cells than the colon, resulting in reduced mucus production. However, Paneth cells are present and produce chemical barrier molecules that help to maintain a separation between intestinal microbes and IECs.

Antimicrobial peptides (AMPs), such as defensins (including α-, β- and θ-defensins) and cathelicidins, are small cationic proteins that disrupt microbial cell membranes. α-defensin (cryptdin) is expressed in Paneth cells in the small intestine and helps protect against pathogenic bacteria (Bosch and Zasloff, 2021; Gong et al., 2021). Meanwhile, cathelicidin-related antimicrobial peptide (CRAMP) is expressed in the colonic epithelia and defends colorectal pathogens (Bosch and Zasloff, 2021). RegIII family proteins are antimicrobial C-type lectins mainly produced by Paneth cells, and they have bactericidal activity against Gram-positive bacteria (Mukherjee et al., 2014). The decrease of this AMP was unveiled to induce peripheral translocation of *Alcanligenes xylosoxidans*, a lymphoid-resident commensal bacteria (Sonnenberg et al., 2012). The expression of RegIII family proteins is controlled by the Toll-like receptor (TLR)/ myeloid differentiation primary response 88 protein (MyD88) pathway and interleukin (IL)-22 stimulation, both of which are activated by the gut microbiota (Frantz et al., 2012; Bhinder et al., 2014). It was observed that the administration of bacterial-associated molecules such as lipopolysaccharide (LPS) or bacterial flagellin could increase the expression of RegIIIγ and enhance the eradication of vancomycin-resistant enterococci (VRE) species (Brandl et al., 2008; Kinnebrew et al., 2010), showing the microbiota can strengthen the gut barrier.

Frantz et al. (2012) showed that mice lacking MyD88 in IECs experienced a decrease in the production of AMPs and mucus, leading to a higher risk of colitis and bacterial infection in the gut. Mice without IL-22 signaling also exhibited increased sensitivity to dextran sulfate sodium (DSS)-induced colitis (Zenewicz et al., 2008), indicating that the compromised chemical barriers in the intestines heighten the susceptibility to inflammation. IL-22 was also revealed to inhibit the growth of segmented filamentous bacteria (SFB), which attached to IECs in the ileum and stimulated the production of serum amyloid A, promoting T helper 17 (Th17) differentiation in the lamina propria (LP) (Terao et al., 2014; Yang et al., 2022). Therefore, IL-22 is capable of preventing Th17-mediated intestinal inflammation.

In addition, Lipocalin 2 (Lcn2), released by immune cells like neutrophils, is a critical antimicrobial protein that sequesters bacterial iron-scavenging siderophores to prevent pathogen iron acquisition (Mayneris-Perxachs et al., 2022). Its expression is induced by inflammation and is dependent on the presence of the gut microbiota (Klüber et al., 2021). Calprotectin, a protein released by neutrophils and IECs during inflammation, also exerts activity against pathogens by sequestering essential divalent metals such as iron, zinc, calcium, and manganese (Zygiel and Nolan, 2018). These are significant for regulating gut microbiota composition and abundance.

#### 3.1.2 Physical barriers

The large intestine, with a higher concentration of goblet cells, has a distinct mucosal barrier system that separates microbiota from the epithelial layer. The mucous layer comprises a firm inner layer and a loose outer layer. The inner layer, consisting of polymerized mucin 2 (MUC2), effectively prevents microorganisms from penetrating the colonic epithelium (Zhang et al., 2021). Transmembrane mucins such as MUC1, MUC13, and MUC17 also protect the intestine from enteric pathogens. Their reduction or deficiency resulted in an increased susceptibility to bacterial infections (van Putten et al., 2017; Sheng and Hasnain, 2022). Furthermore, The *Fut2* gene, responsible for transferring fucose to the end of glycans on cell surface glycoproteins, is linked to an increased susceptibility to Crohn’s disease, a form of inflammatory bowel disease (McGovern et al., 2010; Li L. et al., 2024). A study showed that mice deficient in *Fut2* were significantly more vulnerable to bacterial infections (McGovern et al., 2010), highlighting the key role of *Fut2* in defending against infections.

Various antimicrobial molecules maintain the inner mucous layer in a nearly sterile state, including immunoglobulin A (IgA) and the defensin family of proteins (Palm et al., 2014), while the expression of these antimicrobial molecules in the colon is not as high as in the small intestine due to the absence of Paneth cells and lower IgA^+^ plasma cells (Yanagibashi et al., 2013; Gesualdo et al., 2021). Nonetheless, Ly6/Plaur-domain-containing 8 (Lypd8), a highly N-glycosylated GPI-anchored protein, has been found to efficiently separate intestinal microbes and the epithelium in the colon (Okumura et al., 2016). This suggests that an alternative mechanism is involved in the large intestine to prevent pathogen invasion.

Another physical barrier against the gut microbiota is the cell junctions, specifically tight and adhesion junctions, which connect epithelial cells and control epithelial polarity paralleling the movement of solutes and fluids (Furuse, 2010). These cell junctions build a physical barrier to prevent microbial invasion through the paracellular pathway. Tight junctions comprise claudins, occludins, and intracellular zonula occludens proteins (García-Hernández et al., 2017), which are significant in preventing intestinal inflammation.

Collectively, IECs are essential for the formation and maintenance of the mucosal barrier. They produce barrier components that separate the microbiota from the intestinal immune cells.

### 3.2 Immune-microbiome interaction

Besides the physical separation between the microbiota and the gut, the balanced crosstalk between mucosal immunity and the microbiota is inevitable for sustaining homeostasis in the gut.

#### 3.2.1 Gut-associated lymphoid tissues (GALTs)

GALTs are part of the mucosa-associated lymphoid tissues (MALTs) lining the host and environment. Their primary function is recognizing pathogens, initiating immune response, and maintaining immune tolerance to commensal flora (Mörbe et al., 2021). The histological components of GALTs include Peyer’s patches (PPs), crypt patches, isolated lymphoid follicles (ILFs), appendix, and mesenteric lymph nodes (MLNs) (Mörbe et al., 2021). The formation of GALTs greatly depends on lymphoid tissue inducer (LTi) cells and their interaction with gut microbiota (Jiao et al., 2020). The molecular process involved in this mechanism centers around recognizing pathogen-associated molecular patterns (PAMPs) by pattern recognition receptors (PRRs), which then trigger the activation of downstream signaling pathways. Several key PRR-related molecules have been identified to play roles in this process, including TLR2, nucleotide-binding oligomerization domain 1/2 (NOD 1/2), MyD88, and TIR domain-containing adaptor protein-inducing interferon (IFN)-β (TRIF) (Clarke et al., 2010; Round et al., 2011; Li J.-H. et al., 2024). PRR-PAMP recognition drives the structural development of GALTs and contributes to conditioning the host defense function. Furtherly, metabolic byproducts from symbiotic bacteria, such as short-chain fatty acids (SCFAs), exert effects in modulating the immune response of GALTs through epigenetic mechanisms (Jiao et al., 2020). This helps to maintain immune tolerance towards beneficial microbes.

#### 3.2.2 Innate lymphoid cells (ILCs)

ILCs are vital members of the innate compartment of mucosal immunity (Vivier et al., 2018). ILCs share specific immunological characteristics with Th cells. They are categorized into three significant subpopulations based on their determinant transcription factors and signature cytokines: Group 1 ILCs, like Th1 cells, are T-bet-dependent and secrete interferon gamma (IFN-γ) (Cherrier et al., 2018). Group 2 ILCs, like Th2 cells, are GATA3-dependent and secrete IL-5 and IL-13 (Mjösberg et al., 2012; Roediger et al., 2013). Group 3 ILCs, like Th17 and Th22, are retinoic acid-related orphan receptor (ROR)γt-dependent and secrete IL-17 and IL-22 (Cording et al., 2014; Vivier et al., 2018). Group 3 ILCs consist of the CD4^+^CD3^−^CCR6^+^ subset (LTi cells) and ILC3 subpopulations without expression of the tissue homing factor C-C chemokine receptor 6 (CCR6) (Vivier et al., 2018).

While the ILC1 subset is detectable in germ-free (GF) mice, its number is significantly lower in the fetal intestine, where the gut microbiota is not yet established. This finding may suggest a critical regulatory influence by commensal microorganisms on the maturation and functional manifestation of ILC1 (Krabbendam et al., 2015). ILC1 also contribute to restoring the balance of the intestinal microbiome by inhibiting the growth of opportunistic pathogens like *Clostridium difficile* (Abt et al., 2015). ILC2 are stimulated by cytokines such as IL-25, IL-33, and thymic stromal lymphopoietin that are generated in reaction to signals from commensal bacteria (Peterson and Artis, 2014). It was reported that butyrate, a member of SCFAs, could suppress the production of IL-5 and IL-13 by ILC2 through histone deacetylases (HDAC) inhibition (Thio et al., 2018). ILC3 participate in producing the cytokine IL-22 via the IL-23/IL-23R pathway, promoting the production of antimicrobial compounds like RegIII family proteins by epithelial cells (Dudakov et al., 2015). Meanwhile, ILC3-derived IL-22 and lymphotoxin α (LTα) promote commensal bacteria-dependent expression of *Fut2*, leading to the upregulation of epithelial fucosylation. Fucose generated by commensal bacteria acts as a host defense mechanism against pathogens (Pickard et al., 2014). The interplay between ILC3 and gut microbes is controlled by transcription factors such as inhibitors of DNA binding (ID) 2 and cytokines like IL-22 (Guo et al., 2015; Thaiss et al., 2016). Commensals also modulate the antigen presentation by ILC3, thus shaping the adaptive immune response (Hepworth et al., 2013, 2015). As a result, commensal microbiota is crucial in inducing the host defense function of ILC3.

LTi cells are a unique subset of RORγt^+^ group 3 ILCs that express CCR6 (Huang et al., 2017). The activation of LTi cells is initiated by peptidoglycan derived from enteric Gram-negative commensals, which in turn triggers the activation of stromal cells and leads to the formation of ILFs. This process involves the recruitment of B cells and dendritic cells (DCs) (Bouskra et al., 2008). The microbiota also promotes the production of chemokine (C-C motif) ligand 20 (CCL20) and β-defensin 3, which further activate LTi cells through binding to CCR6 (Bouskra et al., 2008), creating a positive feedback loop for continuous activation and ILFs development.

#### 3.2.3 Intraepithelial lymphocytes (IELs)

IELs are divided into two subsets: natural IELs (nIELs), which migrate directly from the thymus to the intestine, and peripherally induced IELs (pIELs), which differentiate from peripherally activated conventional T cells within the intestinal epithelium (Lockhart et al., 2024). The accumulation of these cell subsets in the intestinal epithelium is closely associated with the diversity of the gut microbiota. nIELs are primarily responsible for tolerance to the indigenous gut microbiota and immune responses against pathogens, whereas pIELs are more involved in immune responses to foreign pathogens. For example, commensal bacteria promote the survival of nIELs through IL-15 produced by IECs, support the cytotoxic potential of TCRαβ^+^ nIELs, and support TCRγδ^+^ nIELs in producing the antimicrobial peptide REGIIIγ (Lockhart et al., 2024). The gut microbiota can regulate pIELs through metabolic products and pattern recognition pathways. *Lactobacillus reuteri*, for instance, produces tryptophan metabolites that induce the expansion of CD4^+^CD8αα^+^ pIELs via the aryl hydrocarbon receptor (AhR) signaling pathway (Cervantes-Barragan et al., 2017). Additionally, *Bifidobacterium* species enhance the accumulation of CD8αβ^+^ pIELs that produce antimicrobial peptides in a TLR-dependent manner (Chen et al., 2018). Members of the *Bacteroidetes* phylum may also promote the development and accumulation of CD4^+^CD8αα^+^ pIELs with anti-inflammatory properties (Bousbaine et al., 2022).

#### 3.2.4 Natural killer T (NKT) cells

NKT cells are a specific T cell subset that share properties of both T cells and NK cells. NKT cells can identify glycolipid antigens presented by CD1d, which is expressed by DCs and IECs (Kayama et al., 2020). It was found that Paneth cells in *Cd1d*-deficient GF mice mono-colonized with *E. coli* showed reduced production of AMPs, leading to their dissemination to MLNs. The fecal microbial community of *Cd1d*-deficient mice was also altered, with increased *Bacteroides* and a lower frequency of *Firmicutes* (Nieuwenhuis et al., 2009). These demonstrate that CD1d-mediated activation of NKT cells is crucial for modulating the colonization, composition, and translocation of commensal bacteria (Nieuwenhuis et al., 2009).

#### 3.2.5 Mucosal-associated invariant T (MAIT) cells

MAIT cells are a specialized subset of T lymphocytes characterized by their semi-invariant T-cell receptors (TCRs), which interact with antigens presented by the major histocompatibility complex (MHC) class I-related molecule 1 (MR1). These cells play a crucial role in the dynamic interaction between the host and the gut microbiota, serving a regulatory function in intestinal immunity by recognizing antigens presented by MR1. MAIT cells are particularly important in defending against bacterial infections; they produce cytokines such as IL-17 and IFN-γ, which enhance mucosal immune responses and contribute to tissue repair (Corbett et al., 2014; Fadlallah et al., 2018). They also recognize acute viral infections by sensing IL-18, IL-15, and type I interferons, and contribute to enhancing the adaptive immune response (Provine et al., 2021). In chronic viral infections like human immunodeficiency virus (HIV) and hepatitis C virus (HCV), a reduction in the number and function of MAIT cells is associated with gut microbiota dysbiosis, which may impair the host’s antimicrobial defenses and the immune system’s recovery (Hengst et al., 2016; Merlini et al., 2019). *Bacteroidetes*, abundant in the gut, are significant stimulants for MAIT cells and may further influence their phenotype in the intestinal mucosa (Tastan et al., 2018). The influence of the microbiota on MAIT cells thereafter highlighted by the fact that certain bacterial metabolites, such as those derived from the riboflavin synthesis pathway by bacteria like *E. coli* and *Proteus mirabilis*, are essential for the development of MAIT cells (Constantinides et al., 2019).

#### 3.2.6 IgA-producing plasm cells

The bacterial symbionts can stimulate the production of IgA in plasm cells (Bunker et al., 2017). PPs are crucial for immune monitoring in the gut, consisting of B cell follicles and T cell zones (Da Silva et al., 2017). DCs are essential for the transportation of antigens from the microbiota to the PPs (Kogut et al., 2020). Within the follicle-associated epithelium of the PPs, specialized epithelial cells called microfold (M) cells contribute to antigen presentation with specific carbohydrates and receptors as binding sites for pathogens (Mabbott et al., 2013; Ohno, 2016). This facilitates the transport of antigens to the subepithelial dome or basolateral pockets where phagocytes, T cells, and B cells reside. These immune cells further stimulate the differentiation of IgA-producing plasma B cells, leading to the production of IgA (Rios et al., 2016; Nagashima et al., 2017). In PPs, follicular helper T (Tfh) cells play a crucial role in orchestrating robust IgA responses by their high expression of the inhibitory coreceptor programmed cell death 1 (PD-1) (Kawamoto et al., 2012). Furthermore, IgA-producing cells are concurrently generated in cecal patches, which are specialized lymphoid tissues found within the appendix. DCs residing within these cecal patches have been shown to potently augment the expression of CCR10 on B cells, thereby ensuring the effective migration of IgA-producing cells to the gut (Masahata et al., 2014; Kayama et al., 2020). IgA is transported through the polymeric immunoglobulin receptor on epithelial cells and released into the intestinal lumen as secretory IgA (SIgA) (Bunker et al., 2015, 2017). SIgA can bind to specific microbial antigens and confine the activity of pathogens (Fagarasan et al., 2010). SIgA is also able to support the survival of the commensal microbes. The absence of IgA results in a reduction in beneficial symbionts and excessive growth of pathogenic bacteria in the intestine (Donaldson et al., 2018; Fadlallah et al., 2018), ultimately leading to intestinal inflammation.

#### 3.2.7 Phagocytes

Intestinal phagocytes mainly include macrophages, DCs, and other non-immune cells. Gut macrophages (Tim-4^−^ macrophages) are derived from circulating monocytes (Shaw et al., 2018). These macrophages are directed to the intestine by the chemokine receptor CCR2, induced by commensal bacteria. The absence of gut microbiota was found to result in a lack of chemotaxis signals for local macrophage replenishment (Lavin et al., 2015). Additionally, a study revealed a positive correlation between myelopoiesis in the bone marrow, the diversity of gut microbiota, and serum TLR levels, suggesting that the resident flora might impact the spatially distant hematopoiesis process through PRR-PAMP pathways (Schürch et al., 2014). Therefore, the absence of commensals can increase susceptibility to bacterial infections due to a compromised immune response mediated by myeloid cells.

DCs in the gut are categorized into two subsets based on the expression of CD103 (αE integrin), a chemokine receptor CX3CR1, and CD11b (Cerovic et al., 2014). In the case of dysbiosis, such as in the *Salmonella* infection model, CD103^+^ DCs were found to accumulate in the enteric epithelium layer and phagocytosed pathogenic bacteria (Farache et al., 2013). Lactic acid bacteria (LAB) were also discovered to stimulate immature DCs within the gut, inducing them to produce cytokines like IL-12 and IL-15 (Rizzello et al., 2011). In the steady state, commensal flora can block the migration of CX3CR1^+^ DCs to MLNs to present both commensal and pathogenic antigens (Diehl et al., 2013), but dysbiosis may disturb this balance and cause inappropriate antigen presentation.

The metabolic products from gut microbiota also impact the local homeostasis of phagocytes and myelopoiesis in the bone marrow (Khosravi et al., 2014). SCFAs can modulate epigenetic processes by directly inhibiting HDACs and activating G-protein coupled receptors (GPCRs) (Woo et al., 2017). Exposure of macrophages to microbiota was reported to result in an elevation of HDAC3, subsequently leading to an increased production of the anti-inflammatory cytokine IL-10. This might be due to the enhanced deacetylation of IL-10 promoters by HDAC3 (Kobayashi et al., 2012). Another study found that SCFAs could affect HDACs levels in gut macrophages, inducing a downregulation of pro-inflammatory cytokines such as IL-6 and IL-12 (Chang et al., 2014). SCFAs were also revealed to support the transformation of macrophages into M2 macrophages, which possess anti-inflammatory properties (Ji et al., 2016). Additionally, Ganal et al. (2012) reported that DCs were unable to produce specific pro-inflammatory cytokines when stimulated by pathogens in GF mice. There was a significant decrease in the level of trimethylated H3K4 in DCs from GF mice (Ganal et al., 2012), suggesting the core role of microbial symbionts or their products in modifying the function of immune cells through epigenetic mechanisms.

In summary, the microbiota is regulated by the barrier system and immune system in the gut. A sustainable mutualism between the host and commensals is necessary for gut homeostasis. The dysfunction of the gut barrier and immune system potentially contributes to the development of infection through the induction of dysbiosis.

## 4 Colonization resistance and invasion

**Figure 3:**
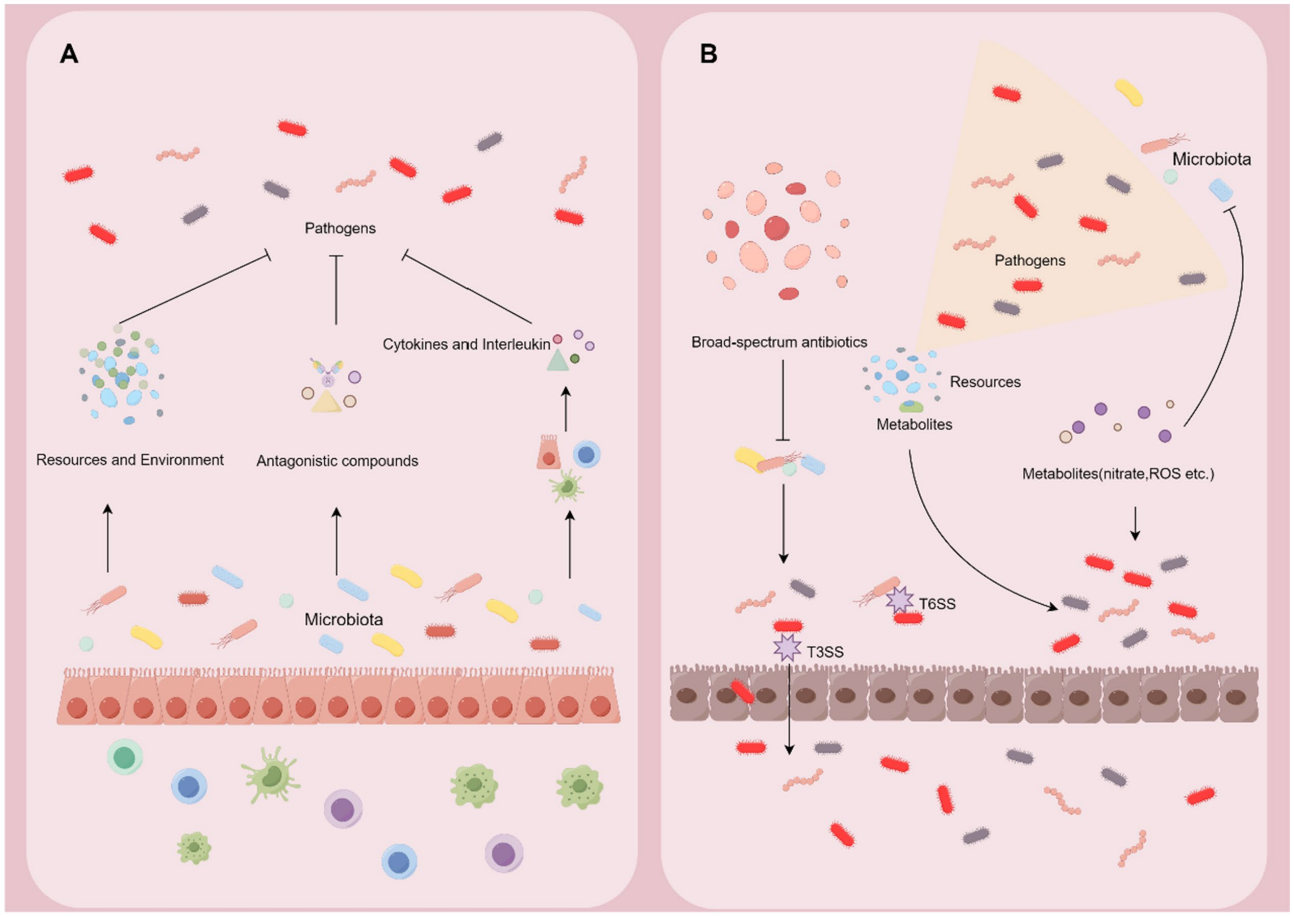
Microbiome-pathogen interaction in the gut. **(A)** Symbiotic microbiota can exert the effect of colonization resistance through altering the resource and environment in the gut, producing antagonistic compounds, and reinforcing the barrier and immune system. **(B)** Pathogens develop counterstrategies including disrupting the microbiota, exploiting nutrients and metabolites, and harnessing intestinal inflammation to break the defense from the microbiota and host.

Colonization resistance refers to the ability of the commensal microbiota to prevent the invasion of pathogens and the overgrowth of indigenous but potentially pathobionts (Caballero-Flores et al., 2023). The microbiota has developed various mechanisms to protect against infection in the gut. However, pathogens evolve strategies to overcome these defenses and thrive (Figure 3).

### 4.1 The competition for resources in the gut

#### 4.1.1 Resource-based colonization resistance

In certain circumstances, the preferential consumption of nutrients in the gut by commensals can outcompete pathogenic microbes. Fabich et al. (2008) showed that *E. coli* competed for nutrients with EHEC, causing starvation of the pathogenic bacteria and thus restricting them. The reduction of dietary amino acids by the microbiota was found to enhance resistance to colonization by the pathogen *Citrobacter rodentium* (Caballero-Flores et al., 2020). Furthermore, more diverse microbiomes increase the probability of protection against pathogens. The overlap in nutrient-utilization profiles between the microbiota community and the pathogen was identified as the critical factor contributing to the benefits of increased microbiome diversity (Spragge et al., 2023). Nutrient utilization assays revealed that beneficial *Klebsiella oxytoca* could utilize 100 different carbon sources, whereas pathogenic *K. pneumoniae* could only utilize 56 carbon sources (Osbelt et al., 2021). Competition for vital metal cofactors such as iron, zinc, and manganese also impacts resistance to pathogenic bateria (Behnsen et al., 2021). These findings emphasize the colonization resistance against pathogens through scrambling for resources in the gut.

#### 4.1.2 Resource-based invasion

Pathogens have numerous strategies to exploit resources in the gut, offering them a competitive edge over commensal microorganisms. They can utilize the molecules that commensal organisms use and alternative nutrient sources. For example, EHEC is able to utilize alternative sources of sugar, such as hexuronate, glucuronate, galacturonate, and sucrose, which are not typically used by commensal *E. coli* (Kamada et al., 2012; Maltby et al., 2013). Moreover, EHEC can use ethanolamine, a carbon and nitrogen source released into the intestine lumen during intestinal cell turnover, as a nutrient based on the *eut* operon in their genome (Garsin, 2010; Girardeau et al., 2011), while commensal *E. coli* cannot utilize ethanolamine as a nutrient. Other pathogenic bacteria, particularly those of food origin, such as *Salmonella Typhimurium* and *Listeria monocytogenes*, have a competitive advantage in the intestines over commensals due to their ability to utilize ethanolamine as an energy source (Girardeau et al., 2011; Thiennimitr et al., 2011). In the case of *Citrobacter rodentium*, it stimulates the expression of genes involved in amino acid biosynthesis to produce its own amino acids, which are crucial for colonizing the gut to overcome the restriction on amino acids imposed by the commensals (Caballero-Flores et al., 2020). This pathogen also shows the ability to utilize alternative carbon sources for its initial proliferation in the gut (Jimenez et al., 2020), efficiently avoiding the limitation of indigenous bacteria. In addition, bacteria in the gut need to acquire iron, which is significant for their growth, relying on the 2,3-dihydroxy benzoate-based siderophore enterobactin (Ent), while innate immune cells produce Lcn2 to inhibit the iron acquisition and growth of commensal bacteria (Khan et al., 2021; Mayneris-Perxachs et al., 2022). However, pathogenic bacteria such as pathogenic *E. coli*, *S. typhimurium*, and *K. pneumoniae* possess a variant of Ent that allows them to evade this inhibition (Fischbach et al., 2006), indicating that some pathogens use nutrients more efficiently than commensals.

Notably, most of the symbiotic bacteria in the intestine are obligate anaerobes. Facultative anaerobe pathogens like *C. rodentium* can take advantage of oxygen availability by attaching to the epithelium and injecting effectors into host cells through Type III secretion systems (T3SS). They change the epithelial metabolism, decrease oxygen consumption, and respire excess oxygen (Lopez et al., 2016; Miller et al., 2020), ultimately enhancing their survival and growth. On the other hand, *C. rodentium* can occupy a unique niche on the intestinal epithelium surface, where commensal bacteria cannot reside, due to its expression of the distinct adhesion molecule intimin (Kamada et al., 2012), enabling *C. rodentium* to avoid competition for nutrients with commensal bacteria.

In general, the nutrient-rich intestine becomes a battleground where commensal and pathogenic microbes compete for dominance by applying various strategies for resources.

### 4.2 The effects of metabolites

#### 4.2.1 Metabolite-based colonization resistance

Commensal microbiota and their metabolic byproducts can create an inhospitable environment for pathogens. The commensals are able to metabolize complex indigestible carbohydrates and mucin into SCFAs, including acetate, propionate, butyrate, valerate, and isovalerate (Nhu and Young, 2023). SCFAs are the primary energy source for colonic enterocytes and support the function of the intestinal barrier system (Fachi et al., 2019; Dang et al., 2023), but they also help resist colonization by pathogens (Osbelt et al., 2020; Pensinger et al., 2023). It was revealed that the administration of *Lactobacillus* to antibiotic-treated mice could result in an increase in fecal butyrate levels and a decrease in intestinal colonization by *K. pneumoniae*, highlighting the defensive function of commensals that produce butyrate (Djukovic et al., 2022). Butyrate was found to reduce the expression of virulence genes in *Salmonella* species, while fucose impacted the expression of virulence factors in enterohaemorrhagic *Escherichia coli* (EHEC) (Gantois et al., 2006; Curtis et al., 2012). Furthermore, the degradation of the primary bile acids in distal intestine by bacteria such as *Clostridium scindens* appeared to inhibit the growth of *C. difficile* and the virulence of *Vibrio cholerae* (Alavi et al., 2020; Foley et al., 2023). The optimum pH is essential for the development of enteropathogenic bacterial species such as *Bacillus cereus*, *E. coli*, and enterotoxigenic bacteria (Ceuppens et al., 2012; Hammami et al., 2013). *Bacteroides* were reported to resist colonization by changing the gut pH through the production of propionate (Brinkman et al., 2013), indicating that the SCFAs from commensals might help influence the pH of the environment, thus altering the physiological conditions required for pathogen virulence and survival.

The immune system can also be regulated by the metabolites from gut microbiota to resist pathogens. Pedicord et al. (2016) discovered that the commensal bacterium *Enterococcus faecium* could protect against *S. typhimurium* infection by producing secreted antigen A (SagA), a distinct peptidoglycan hydrolase. SagA can interact with PRRs in the IECs, leading to a strong innate immune response against pathogens (Pedicord et al., 2016). In a mice model of sepsis, fecal microbiota transplant (FMT) was found to improve pathogen clearance and restore host systemic immunity. This beneficial effect of FMT was associated with an increase in butyrate-producing *Bacteroidetes*, which enhanced the expression of interferon regulatory factor (IRF) 3, ultimately alleviating inflammation (Kim et al., 2020). The establishment of a specific *Bacteroidota* consortium in GF and antibiotic-treated mice could confer protection against *K. pneumoniae* by activating IL-36 signaling pathways (Sequeira et al., 2020), resulting in increased resistance to enteric pathogens. Furthermore, colonization of GF mice with commensal bacteria has been discovered to induce the presence of Th17 in the intestinal tissue (Ivanov et al., 2009), suggesting the modulation of microbiota on immune cells.

In addition, specific microbes can produce compounds that have antagonistic effects on pathogens. For example, substances with inhibitory effects on VRE include a lantibiotic produced by *Blautia producta* and bacteriocin produced by *Enterococcus faecalis* (Kommineni et al., 2015; Kim et al., 2019). *Lactobacillus lactis* and *Streptococcus* can produce Nisin-A, which is widely utilized as a food preservative due to its inhibiting the growth of gram-positive bacteria (Nhu and Young, 2023).

Commensal bacteria also demonstrate the ability to limit the colonization of pathogenic microorganisms other than bacteria by producing specific metabolites. *C. scindens* and *Clostridium orbiscindens* were discovered to exert protective effects against the Chikungunya and influenza viruses in mice, respectively. The mechanism of action for *C. scindens* involves the enhancement of the antiviral immune response through the production of deoxycholic acid. On the other hand, *C. orbiscindens* and its metabolite desaminotyrosine were reported to modulate type I IFN signaling, efficiently restraining influenza virus (Steed et al., 2017; Winkler et al., 2020). Fan et al. (2015) illustrated the critical role of commensal anaerobic bacteria, specifically clostridial *Firmicutes* (clusters *IV* and *XIVa*) and *Bacteroidetes*, in maintaining resistance to *Candida albicans* colonization in mice. This finding emphasized the significance of hypoxia-inducible factor-1α (HIF-1α) and the antimicrobial peptide LL-37 in determining the effect to resist *C. albicans* colonization (Fan et al., 2015). Another study using a mice model of parasitic protozoa revealed that *C. scindens* served a protective role against *Entamoeba histolytica* infection (Burgess et al., 2020). This protective mechanism relied on the metabolism of bile salts, as the bile salt-derived metabolite deoxycholate was found to activate the host bone marrow through epigenetic modifications, thereby boosting the immune response (Burgess et al., 2020). It is also evident that the commensal *E.coli* express anti-α-gal antibodies, which confer protection against *Plasmodium* species infection (Yilmaz et al., 2014; Mandal and Schmidt, 2023), thus restricting the transmission of malaria parasites. These findings underscore the potential of gut microbiota and their metabolites to resist viral, fungal, and parasitic invasion, providing novel therapeutic options for treating these infections.

#### 4.2.2 Metabolite-based invasion

The pathogen can take advantage of the metabolites produced by beneficial bacteria. The gut is rich in sugar molecules broken down by saccharolytic bacteria (Horrocks et al., 2023). It was found that *Bacteroides thetaiotaomicron* could break down sugar components, such as sialic acid and fucose, from the gut epithelial mucins. These sugar components could then be utilized by pathogenic bacteria such as *C. difficile* and *S. typhimurium* (Wang et al., 2013). In addition, *S. typhimurium* was exhibited to utilize gut microbiota-derived hydrogen to fuel its early-stage growth and expansion during infection (Maier et al., 2013). Taurocholate and cholate, generated by commensals, were reported to be potent triggers for the germination of *C. difficile* spores in the gut (Aguirre et al., 2021; Foley et al., 2023). A recent study revealed that the presence of the specific gut microbiota was able to resist *Campylobacter jejuni*, mainly because the microbiota rarely produced organic acids and amino acids, which were vital for *C. jejuni* growth (Shayya et al., 2023). These findings show that pathogens can effectively utilize the metabolites of resident bacteria as a source of energy to promote their growth.

Some pathogenic microbes rely on the molecules produced by microbiota as metabolic signals to promote their activity. For example, EHEC uses fucose as a signaling molecule to modify their metabolism and gene expression related to virulence and metabolic stimulus. This is achieved through a fucose-sensing signaling transduction system composed of FusK and FusR components, helping EHEC adapt to the intestinal lumen environment (Curtis et al., 2012). Similarly, ethanolamine acts as a signal molecule for EHEC and *S. typhimurium*, triggering the expression of their virulent genes (Kendall et al., 2012; Anderson et al., 2015). *C. difficile* can utilize microbiota-produced succinate to gain a growth advantage in the gut (Ferreyra et al., 2014). Regarding SCFAs, the variation of their distribution, concentration, and composition in the intestine results in the creation of distinct physiological environments sensed by pathogenic bacteria. It was observed that the concentration of acetate in the ileum region of the intestine enhanced the expression of pathogenicity island 1 (SPI-1)-encoded T3SS of *S. typhimurium*, promoting bacterium invasion (Khan et al., 2021). In contrast, propionate and butyrate in the colon were found to repress T3SS-1-related genes (Maurer et al., 2002). In the case of EHEC, T3SS expression was elevated by butyrate concentration in the colon via post-transcriptional activation of Lrp, a transcriptional regulator in EHEC (Takao et al., 2014). However, exposure to acetate and propionate in the small intestine was not evident to affect gene expression related to EHEC virulence or T3SS (Takao et al., 2014), showing that the susceptibility to infection may influenced by the metabolites of commensals.

The pathogens are also able to utilize the host immune response to gain a growth advantage over symbiotic microbes in the gut through their metabolism. Inflammation can lead to changes in the intestinal environment, creating an environment more conducive to the growth of pathogens (Fattinger et al., 2021). For example, pathogenic bacteria such as EHEC are facultative anaerobes and can use nitrate as an energy source (Lee et al., 2022). During intestinal inflammation, nitrate (NO3^−^) production is increased in the intestine due to the migrated neutrophils and macrophages with inducible nitric oxide synthetase (iNOS). Pathogenic bacteria can apply the nitrate as an energy source through nitrate respiration, while commensal bacteria lack this ability (Winter et al., 2010). The cytokine IFN-γ also stimulates the production of hydrogen peroxide and nitric oxide in the gut, which can be converted into nitrates (Khan et al., 2021). Similarly, the influx of neutrophils during inflammation produces reactive oxygen species (ROS), which are responsible for converting S_2_O_2_^−3^ generated by commensal bacteria into S_4_O_2_^−6^. This form of sulfur cannot be utilized by symbionts but by pathogens like *S. typhimurium* through its specific operon *ttrSR ttrBCA* (Furne et al., 2001; Winter et al., 2010).

Furthermore, intestinal inflammation serves as a signal that triggers and amplifies the expression of virulence factors in the pathogens. A study (Licheng et al., 2005) presented that *Pseudomonas aeruginosa*, a bacterium that frequently caused infections in hospital settings, led to nosocomial infection by using its surface protein OprF to bind to the host immune factor IFN-γ. This interaction further activated a quorum sensing-dependent virulence factor PA-I lectin (Licheng et al., 2005). Additionally, IL-22 was exhibited to be abundant during *S. typhimurium* infection and correlated with high levels of galactoside 2-α-l-fucosyltransferase 2. This enzyme could promote the α(1,2)-fucosylation of mucus carbohydrates, activating fucose-related genes in other pathogenic members such as EHEC (Tu et al., 2014). The increase in mucus-derived carbohydrates in the gut lumen establishes a physiological niche enriched with pathogen-specific nutrients, altering the composition of the gut microbiota and potentially contributing to infection (Sonnenburg et al., 2005; Wang et al., 2013). Consequently, the ability to subvert colonization resistance and thrive in an inflammatory environment is a critical factor in the pathogenicity of many infectious agents.

### 4.3 Contact-dependent interactions

Some competitions between commensal microbes and pathogens rely on direct cell–cell contact. For indigenous microbiota, contact-dependent inhibition requires a specific receptor protein on the target cell and the encoding of various toxic effector domains with inhibitory mechanisms (Hayes et al., 2014; Wang et al., 2024). Commensal species like *E. coli* are equipped with type VI secretion systems (T6SS), a contractile nanomachine widely distributed among Gram-negative bacteria, which allow them to directly inject antimicrobial substances into their competitors like *C. rodentium*, efficiently neutralizing them and preventing the colonization (Flaugnatti et al., 2021; Serapio-Palacios et al., 2022). Furthermore, the probiotic *Bacillus* demonstrates the effect of influencing the quorum-quenching fengycins, previously known for their anti-fungal properties, effectively eradicating *Staphylococcus aureus* infection by interfering with its signaling mechanism (Piewngam et al., 2018; Su and Ding, 2023). These advanced defense strategies testify to the adaptability and complexity of bacterial survival mechanisms. However, the T6SS is also used by pathogens to compete with the native microbiota by injecting toxic effectors into nearby cells. Pathogens like *V. cholerae*, *S. typhimurium*, *Shigella sonnei*, and *C. rodentium* can apply their T6SS to kill competing beneficial microbes (Sana et al., 2016; Anderson et al., 2017; Zhao et al., 2018; Serapio-Palacios et al., 2022), thereby promoting their colonization in the gut.

### 4.4 Disruption of microbiota

The dysbiosis can lead to enteric infections originating from the expansion of indigenous but potentially pathogenic microbes. Numerous factors, including host genetics, diet, and the use of antibiotics, can influence the composition and functionality of the gut microbiota (Pickard and Núñez, 2019; Qin et al., 2022). The disruption of the gut microbiota community reduces beneficial bacteria diversity or leads to an outgrowth of pathobionts, making the host more vulnerable to opportunistic infections caused by potential pathobionts (Gupta and Dey, 2023). *C. difficile* infection is a leading opportunistic infectious disease associated with diarrhea and colitis (de Nies et al., 2023). Under homeostatic conditions, the growth of *C. difficile* is controlled by commensals; however, broad-spectrum antibiotic treatment is able to induce *C. difficile* infection with the production of toxins TcdA and TcdB (Vuotto et al., 2024). Similarly, *S. typhimurium* and *E. coli* are bacterial enteric pathogens commonly linked to the consumption of contaminated food. The disruption of commensal microbiota promotes the growth of these pathogens and triggers inflammation (de Nies et al., 2023). The administration of broad-spectrum antibiotics can also create opportunities for the overgrowth of resistant pathogens (Diaz Caballero et al., 2023; Yip et al., 2023), leading to the development of difficult superinfections to treat.

Noteworthy, it was presented that gut fungal dysbiosis reduced the efficacy of FMT for treating recurrent *C. difficile* infections (Zuo et al., 2018), highlighting the interdependencies between bacteria and fungi in the GI tract. Antibiotic treatments can result in fungal overgrowth by reducing competing bacteria, while antifungal treatments can alter bacterial communities (Wheeler et al., 2016; Sovran et al., 2018). Fungi can act as a commensal that protects the host from bacterial pathogens by modulating the immunological responses of the host (van Tilburg Bernardes et al., 2020; Pérez, 2021). For example, *Candida albicans* influence host immune responses, especially Th17, which are crucial for protective immunity at barrier sites (Bacher et al., 2019). Conversely, indigenous bacteria can effectively limit the growth and invasion of fungal pathogens by targeting specific virulence factors, disrupting quorum sensing systems, secreting active metabolites, or triggering the immune response (Cong et al., 2023). Therefore, the interplay between bacteria and fungi within the gut microbiota substantially influences its balance.

Collectively, gut commensal microbes effectively exert robust resistance against pathogen colonization through diverse mechanisms. These mechanisms mainly include competition for resources, utilization of metabolic by-products, and direct contact suppression. In response, pathogens have evolved strategies to evade defenses, resulting in dysbiosis and infection. To further enhance colonization resistance, advanced therapies based on microbiota have been introduced to prevent and treat infections in the GI tract.

## 5 Microbiota-targeted therapy

For years, microbiota-targeted therapy has been employed to prevent and eradicate enteric infections (Sorbara and Pamer, 2022; Fekete et al., 2023). This specialized therapy involves a variety of approaches.

One approach is with FMT. The transplantation of beneficial microbiota helps restore the diversity and stability of the GI microbial community, protecting against the overgrowth of pathogenic microbes. FMT efficiently treats recurrent *C. difficile* infection (CDI), which is often refractory to conventional antibiotic therapy (Gupta et al., 2022; Gonzales-Luna et al., 2023). A study demonstrated that the administration of a consortium of five special strains effectively limited VRE gut colonization in antibiotic-treated mice by depleting nutrients, particularly fructose (Isaac et al., 2022). This provides a new strategy for applying FMT to treating enteric infections caused by various pathogens. FMT can be performed through multiple routes, such as oral capsules, enemas, or colonoscopy, depending on the severity of the infection and the patient’s preference (Liwinski and Elinav, 2020). Nonetheless, further research is necessary to determine suitable donors for FMT, the most effective administration methods, and the pivotal factors influencing therapeutic effectiveness. Personalized approaches that consider the unique composition of gut microbiota and immune system in each individual are crucial for maximizing the efficacy of FMT.

Another approach is the administration of probiotics. Probiotics are live microorganisms that provide benefits to the host. These microorganisms can act by directly inhibiting the growth of pathogens, enhancing the immune response, and producing antimicrobial substances (Zhong et al., 2022). Furthermore, prebiotic supplementations are dietary compounds that selectively promote the growth and activity of beneficial microorganisms in the gut. They serve as a source of nutrients for these microbes, thus indirectly enhancing the colonization resistance. Prebiotics can be found in various foods such as fruits, vegetables, whole grains, and legumes (Sanders et al., 2019). The application of prebiotics and probiotics in treating enteric infections requires careful consideration of the specific strains and doses to ensure their efficacy. Understanding the metabolic pathways commensal microbiota uses for infection prevention will aid in developing next-generation prebiotics and probiotics with enhanced anti-pathogenic capacity. However, in some instances, it may be more beneficial to restrict bacterial nutrients rather than adding a prebiotic. This can be achieved through dietary changes, such as following a low fermentable oligosaccharides, disaccharides, monosaccharides, and polyols (FODMAP) diet (McCuaig and Goto, 2023). By removing the resource in the gut, the microbial community is altered, highlighting the impact of nutrient restriction on microbial composition.

Additionally, there has been a remarkable advancement in the application of small microbial molecules and engineered bacteriophages for the targeted eradication of specific pathogens. Small microbial molecules, such as butyrate, produced by beneficial microorganisms, act as supplements to overcome the challenges associated with engrafting live microorganisms (Chen et al., 2019). Engineered bacteriophages, which are viruses that specifically target and kill bacteria, can selectively eliminate pathogenic bacteria while sparing the beneficial ones (Divya Ganeshan and Hosseinidoust, 2019; Jault et al., 2019). The potential of harnessing bacteria that produce AMPs to enhance colonization resistance is also worth investigating further.

The integration of emerging metagenomics, novel imaging technologies, and mathematical modeling potentially contributes to precision microbiome reconstitution therapy in the gut (Bilotta and Cong, 2019; Chetty and Blekhman, 2024). Further investigation is essential to uncover novel and unique therapeutic targets within the microbiota which can be utilized for the precise prevention and management of enteric infections.

## 6 Conclusion

This review mainly discusses about two crucial interactions involving microbiota in the gut. The first is the crosstalk between the host and microbial symbionts, while the second is the constant battle between the commensal microbiota and pathogens. The microbiota serves as an indispensable mediator in these interactions, orchestrating the gut ecosystem and the resistance to pathogen colonization, whereas dysbiosis can lead to enteric infection. Therapies focusing on the gut microbiota primarily aim to restore balance in the indigenous symbionts and enhance their protective functions. A deeper understanding of the mediating roles of microbiota is vital to providing effective and personalized treatments for enteric infections.

## Author contributions

YC: Writing – original draft, Writing – review & editing. LX: Writing – original draft, Writing – review & editing. MZ: Writing – review & editing. HZ: Writing – review & editing.
